# Comparison of the effect of hostility on the level of depression of drug addicts and non-addicts and the mediating role of sense of life meaning between them

**DOI:** 10.1186/s12888-023-04856-z

**Published:** 2023-05-21

**Authors:** Jiaoyang Li, Rufang Wang, Jingzhen He, Linghui Wang, Lin Li

**Affiliations:** 1 Chengdu University of Traditional Chinese Medicine, 1166 Liutai Avenue, Wenjiang District, Chengdu, China; 2 The Second Drug Rehabilitation Center in Chengdu, Chengdu, China; 3 The Drug Rehabilitation Center in Chengdu, Chengdu, China

**Keywords:** Drug addicts, Non-addicts, Hostility, Depression, Sense of life meaning

## Abstract

**Background:**

The level of depression among drug addicts is generally higher than normal. Hostility and sense of life meaning may influence depression and become risk factors for depression. This study has three research purposes. First, to analyze whether drug use can aggravate hostility and depression levels. Second, to assess whether the hostility has different effects on depression among drug addicts and non-addicts. Third, to examine whether the sense of life meaning has a mediating role between different groups (drug addicts and non-addicts).

**Methods:**

This study was conducted from March to June 2022. 415 drug addicts (233 males and 182 females) and 411 non-addicts (174 males and 237 females) were recruited in Chengdu, Sichuan Province. After signing informed consent, their psychometric data were obtained using the Cook-Medley Hostility Scale (CMI), Beck Depression Inventory (BDI) and Meaning in Life Questionnaire (MLQ) questionnaires. Linear regression models were used to assess the impact of hostility and depression among drug addicts and non-addicts. Bootstrap mediation effect tests were used to further test the mediation effect of sense of life meaning between hostility and depression.

**Results:**

The results showed four main outcomes. First, compared with non-addicts, drug addicts had higher levels of depression. Second, hostility exacerbated depression in both drug addicts and non-addicts. Compared with non-addicts, hostile affect had a greater effect on depression in drug addicts. Third, the sense of life meaning among females was higher than males. Fourth, for drug addicts, the sense of life meaning showed a mediating effect between social aversion and depression, while for non-addicts, the sense of life meaning showed a mediating effect between cynicism and depression.

**Conclusions:**

Depression is more severe in drug addicts. More attention should be paid to the mental health of drug addicts, because the elimination of negative emotions is conducive to reintegration into society. Our results provide a theoretical basis for reducing depression among drug addicts and non-addicts. As a protective factor, we can reduce their hostility and depression by improving the sense of life meaning.

## Study design

Hostility is a risk factor for depression, and the sense of life meaning is also an important factor for depression. The purpose of this paper is to investigate how hostility affects the degree of depression and the mediating role of the sense of life meaning between them.

## Introduction

Drug addiction is a serious global health problem caused by physical, psychological and social disorders, and it is a major factor in increasing suicide risk [1]. In a record analysis, 43.9% of drug addicts had suicidal behaviors [2]. According to the United Nations Office on Drugs and Crime World Drug Report 2022, approximately 300 million people around the world took drugs this year, making drug use a global issue. In June 2022, the Chinese government website released “Drug Situation in China (2021)”, which showed that there were 1.486 million drug addicts nationwide. Although it decreased by 17.5% compared to last year, the drug situation was still severe. In China, the pooled prevalence of illicit drug use was about 2.10%, and Sichuan showed a high prevalence [3]. A study showed that 83% of drug addicts were addicted to multiple drugs, and 68% of drug addicts preferred oral administration, while 32% used drugs through a combination of oral, inhalation, and injection routes [4]. At the same time, multiple prospective studies have shown that drug addiction is strongly related to mental problems [5, 6], including depression and anxiety [7], and these psychiatric problems and drug addiction are common comorbidities in psychiatry.

Hostility is a negative attitude of antagonism and hatred towards others, characterized by distrust, irritability [8], and high aggression [9]. The severe hostility of drug addicts can lead to extremely autonomous and aggressive behaviors that are not conducive to social stability. Criminal tendencies are often associated with violent behaviors among drug addicts, and 39.68% of drug addicts have a history of violent behaviors [10], with the prevalence of violent behaviors among female drug addicts significantly higher than among male drug addicts (63.3% vs. 24.2%) [11]. Such people are vulnerable to discrimination and unfair treatment in society, which in turn increase their level of hostility. Several studies have shown that hostility is associated with physical ailments [12–14], such as heart disease and chronic obstructive pulmonary disease, which affect the physical and mental health of individuals. Depression is usually a negative emotion that has been defined as persistent sadness, hostility and irritability [15]. Studies have shown that drug addicts have higher levels of depression than non-addicts (Mean = 32.70 vs. 17.77) [16]. The prevalence of depression among addicts is high [17]. One study showed that the incidence of severe depression among drug addicts was 44%, while the incidence of mild to moderate depression was 25% [18]. Studies have found that 57.6% of the participants showed any type of mental symptoms, including depression, anxiety and psychotic symptoms [19]. It can be seen that a larger proportion of drug addicts suffer from mental illness such as depression. At the same time, negative emotions such as depression are important reasons for drug addiction [20, 21], and negative emotions of depression increase the likelihood of relapsing [22]. In addition, some researchers have found a strong association between hostility and depressive disorder individuals with a high level of hostility having a higher level of depression [23]. At the same time, individuals with severe depression also showed a higher level of hostility [24]. According to previous studies, drug addicts generally have a high level of depression [25]. In terms of gender differences in depression, psychologically, more females are depressed than males [26]. A study of depression in adolescents found that common environmental factors had greater impacts on adolescent girls’ versus boys’ depressive symptoms (13% vs. 1%) [27].

Sense of life meaning was defined by Steger as a positive variable, which was the perception of and search for purpose and value in one’s life [28]. One study showed that the sense of life meaning was lower among drug addicts [29]. A strong relationship has been documented between the sense of life meaning and depression in both high and low mental states [30]. Researchers have shown that an increased sense of life meaning was associated with reduced depression [31]. Episodes of depression are associated with a low level of life meaning and a high level of hostility. Therefore, more attention should be paid to the sense of life meaning of drug addicts. Researchers have included the sense of life meaning into the presence of meaning and the search for meaning [28]. Various studies have confirmed that the two dimensions of sense of life meaning have different effects on psychological problems. The presence of meaning is negatively correlated with psychological problems such as depression [32], while the search for meaning is positively correlated with depression [33]. That is, positive life meaning helps maintain mental health. This study focuses on the negative relationship between sense of life meaning and depression, and examines how the presence of meaning affects depression.

Reducing depressive states is of great importance in promoting physical and mental health. However, few studies have investigated the relationship between sense of life meaning and hostility. And there are limited studies in special populations and gender differences, especially for drug addicts. It is also worth exploring whether the sense of life meaning can be a way to regulate hostility and improve depressive states. Therefore, there is a need to further discuss the role of sense of life meaning in the relationship between hostility and depression. Increasing the sense of life meaning is a guide to reducing depression levels among drug addicts, promoting effective drug prevention and improving social stability.

## Methods

### Design

The study was conducted from March to June 2022, and participants were informed of the purpose and confidentiality of their participation before the investigation. After signing informed consent, participants completed the Cook-Medley Hostility Scale (CMI), Beck Depression Inventory (BDI), and Meaning in Life Questionnaire (MLQ) questionnaires under the guidance of a professional instructor. We obtained research data through questionnaires completed by participants and distributed souvenirs and gifts to all participants.

### Participants

Using simple random sampling to recruit participants. A total of 826 participants were investigated in this study. These participants were divided into two groups: drug addicts (233 males and 182 females) and non-addicts (174 males and 237 females). These addicts were recruited from two drug rehabilitation centers in Sichuan Province, China. Among drug addicts, about 82% were abstainers who used methamphetamine, while the rest used heroin, marijuana, and ketamine. The entry criteria for drug addicts were as follows: (a) Drug addicts who have completed physical detoxification and have a negative urine test. (b) Meeting the DSM-V diagnostic criteria for psychoactive substance abuse or dependence. (c) Absence of serious mental illness and not taking any medication, (d) Age 16–65 years old, and (e) Education background of elementary school or above.

Non-addicts (174 males and 237 females) were some college students and people employed in Sichuan Province, China. The entry criteria for non-addicts were as follows: (a) Absence of serious mental illness and not taking any medication, (b) Age 16–65 years old, and (c) Education background of elementary school or above. The purpose of the study was explained to the participants, and all participants signed informed consent before participation.

### Measures

#### Cook-Medley Hostility Scale

The Cook-Medley Hostility Scale (CMI) was an indicator of health developed by Cook and Medley [34]. The scale used in this research covered the 43-item subset determined by John C. Barefoot [35], which included five dimensions, namely, cynicism, hostile affect, aggressive response, hostile attitude, and social aversion, with a total of 43 items. Higher total scores on the CMI indicated a higher level of hostility. In this study, the Cronbach’s Alpha value of CMI was 0.769 in drug addicts and 0.805 in non-addicts.

#### Beck depression inventory

The Beck Depression Inventory (BDI) was written by Beck in 1996 [36] and was used to assess the severity of depressive symptoms within two weeks. This scale consisted of 21 items, each rated from 0 to 3. Higher total scores indicated a higher level of depression. In this study, the Cronbach’s Alpha value of BDI was 0.854 in drug addicts and 0.909 in non-addicts.

#### Meaning in life questionnaire

The Meaning in Life Questionnaire (MLQ) was written by Steger in 2006 [28] and emphasized the meaning in one’s life. The MLQ included two dimensions: the presence of meaning and the search for meaning. The items of the MLQ were rated from 1 to 7, with higher total scores indicating a higher level of sense of life meaning. In this study, the Cronbach’s Alpha value of MLQ was 0.754 in drug addicts and 0.830 in non-addicts.

#### Statistical analysis

We used SPSS 25.0 and AMOS 24.0 for data entry, collation, and analysis. First, Harman’s one-factor test was used to test for common method bias. Second, descriptive statistics were used to analyze the demographic information of drug addicts and non-addicts. Moreover, measures such as age, BMI, CMI, BDI and MLQ among drug addicts and non-addicts were described by means and standard deviations (M ± SD) and compared by t-test. Third, Pearson correlation analysis was used to assess the correlation between variables. Fourth, linear regression models were used to assess the impact of hostility and depression among drug addicts and non-addicts. Meanwhile, gender, age, and BMI were controlled as covariates. Finally, we used AMOS 24.0 to establish intermediary models and used bootstrap mediation effect tests to further test the mediation effect of sense of life meaning. Statistical significance required a two-sided *P*-value of ≤ 0.05.

## Results

### Common method bias test

The data from this study were tested for common method bias using Harman’s one-factor test, as recommended by Podsakoff [37]. The results showed that there were 21 factors with a characteristic root greater than 1, and the variance of the maximum factor interpretation was 11.812%, which was less than 40% of the critical standard. This finding indicated that there was no common problem of methodological bias in the questionnaire used in this study.

### Sample description

Table 1 shows the gender and group differences in demographic characteristics and scale-related dimensions between drug addicts and non-addicts. There were significant gender and group differences in age and BMI between drug addicts and non-addicts. Cynicism, hostile affect, hostile attitude had significant group differences. Non-addicts had significantly lower BDI than drug addicts. Female had significantly higher MLQ than male.

**Table 1:** Gender and group differences in demographic characteristics and scale-related dimensions (*n* = 826)

Characteristics (M ± SD)	Drug addicts		Non-addicts		p	
	Male (*n* = 233)	Female (*n* = 182)	Male (*n* = 174)	Female (*n* = 237)	P(gender)	P(group)
Age	39.30 ± 9.82	33.63 ± 7.69	23.99 ± 7.96	22.06 ± 6.16	< 0.001	< 0.001
BMI kg/m^2^	22.87 ± 3.03	23.37 ± 3.16	21.65 ± 2.92	20.51 ± 2.68	0.007	< 0.001
Cynicism	6.24 ± 2.57	5.19 ± 2.37	5.14 ± 2.49	5.33 ± 2.48	0.004	0.002
Hostile affect	2.28 ± 1.18	2.17 ± 1.14	2.36 ± 1.32	2.66 ± 1.22	0.123	< 0.001
Aggressive response	3.94 ± 1.95	3.58 ± 1.61	3.47 ± 1.99	3.57 ± 1.74	0.199	0.05
Hostile attitude	5.10 ± 2.23	4.75 ± 2.18	4.57 ± 2.26	4.30 ± 1.98	0.011	< 0.001
Social aversion	1.78 ± 1.04	1.67 ± 0.95	1.67 ± 1.17	1.74 ± 1.08	0.778	0.766
BDI	16.71 ± 10.03	12.49 ± 7.81	10.94 ± 10.50	9.23 ± 7.56	< 0.001	0.001
MLQ	45.91 ± 9.81	48.90 ± 9.28	47.46 ± 8.07	47.81 ± 9.31	0.008	0.492

P(gender): "gender" means that all participants were grouped by gender, P(group): "group" means that all participants were grouped by whether addicts or not

*BDI:* Beck Depression Inventory, *MLQ:* Meaning in Life Questionnaire

### Correlational analysis

Correlations between all variables are presented (Table 2). CMI (cynicism and social aversion) and MLQ were negatively correlated (*P* < 0.01). Meanwhile, CMI (cynicism, hostile affect, aggressive response, hostile attitude, and social aversion) and BDI were positively correlated (*P* < 0.01), whereas MLQ was negatively correlated with BDI (*P* < 0. 01).

**Table 2:** Correlations among CMI, BDI, and MLQ

	1	2	3	4	5	6	7
1. Cynicism	1						
2. Hostile affect	0.366**	1					
3. Aggressive response	0.416**	0.338**	1				
4. Hostile attitude	0.498**	0.330**	0.443**	1			
5. Social aversion	0.328**	0.332**	0.298**	0.282**	1		
6. BDI	0.260**	0.131**	0.119**	0.306**	0.187**	1	
7. MLQ	-0.107**	-0.06	-0.006	-0.065	-0.118**	-0.255**	1

^**^ *P* < 0. 01

### Effects of CMI on BDI among drug addicts and non-addicts

Table 3 shows the effects of the five dimensions of CMI on BDI of drug addicts and non-addicts. This table presents the beta coefficients and standard errors from regression models in which BDI scores were regressed on one CMI, controlling for gender, age, and BMI. The results showed that five dimensions of CMI were significantly correlated with BDI in non-addicts. Among drug addicts, when compared with non-addicts, hostile affect had a greater impact on the degree of depression (beta coefficient: 0.209 vs. 0.142). However, cynicism, aggressive response, hostile attitude, and social aversion were more impactful on the degree of depression of non-addicts.

**Table 3:** Effects of CMI on BDI among drug addicts and non-addicts

Variables	Drug addicts			Non-addicts		
	β ± SE	T	P	β ± SE	T	P
Cynicism	0.192 ± 0.048	4.013	< 0.001	0.257 ± 0.048	5.402	< 0.001
Hostile affect	0.209 ± 0.047	4.474	< 0.001	0.142 ± 0.049	2.880	0.004
Aggressive response	0.078 ± 0.048	1.635	0.103	0.123 ± 0.049	2.507	0.013
Hostile attitude	0.229 ± 0.047	4.912	< 0.001	0.317 ± 0.047	6.714	< 0.001
Social aversion	0.179 ± 0.047	3.776	< 0.001	0.201 ± 0.048	4.163	< 0.001

All the regression coefficients in the table are standardized regression coefficients

### Structural equation modeling

**Fig. 1:**
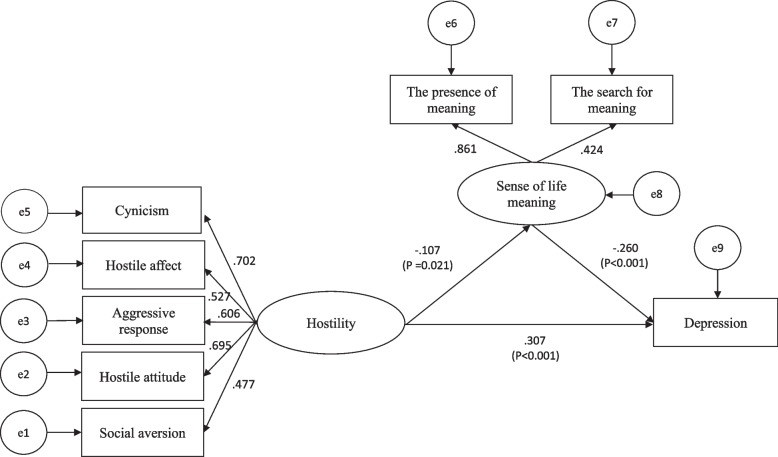
Mediating role of sense of life meaning

The structural equation model shown in Fig. 1 was constructed to predict whether there was a mediating effect between hostility and depression. Amos 24.0 was used to test the mediation model, and the fitting index of the whole model was obtained as follows:χ^2^/df = 4.100, RMSEA = 0.061, IFI = 0.932, GFI = 0.974, AGFI = 0.953, CFI = 0.932. These values demonstrated that this structural equation model fits the data well. Hostility was positively associated with depression (β = 0.307, t = 7.033, *p* < 0.001) and was negatively associated with sense of life meaning (β = -0.107, t = -2.299, *p* = 0.021). Further, the sense of life meaning was negatively associated with depression (β = -0.260, t = -3.616, *p* < 0.001). Therefore, hostility can affect depression in drug addicts and non-addicts through the sense of life meaning.

The path coefficients in the model were further analyzed. Sense of life meaning played a partial mediating role between hostility and depression (indirect effect β = 0.058, *p* = 0.021, direct effect β = 0.640, *p* = 0.001, total effect β = 0.698, *p* < 0.001). Indirect effects accounted for 8.3% of the total effects.

### The mediating role of the presence of meaning

**Fig. 2:**
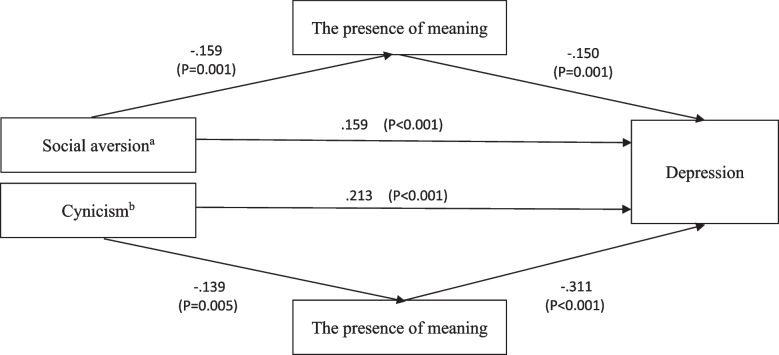
The structural equation model of the relationship between hostility and depression. Note. ^a^: social aversion of drug addicts; ^b^: cynicism of non-addicts

The structural equation model shown in Fig. 2 was constructed to predict how the presence of meaning mediates the relationship between hostility and depression and the mediating effects of different groups. For drug addicts, the presence of meaning displayed a mediating effect between social aversion and depression (indirect effect β = 0.024, *p* = 0.001, direct effect β = 0.157, *p* = 0.003, total effect β = 0.181, *p* < 0.001), which accounted for 13.3% of the total effect. For non-addicts, the presence of meaning displayed a mediating effect between cynicism and depression (indirect effect β = 0.043, *p* = 0.004, direct effect β = 0.214, *p* < 0.001, total effect β = 0.257, *p* < 0.001), which accounted for 16.7% of the total effect. In addition, the mediation model with other dimensions of hostility in drug addicts and non-addicts has no significant mediation effect.

## Discussion

### Hostility aggravated depression among drug addicts and non-addicts

Existing research has shown a unique relationship between hostility and major depression—that is, higher level of hostility was correlated with more severe cases of depression [38], while depressed individuals showed greater hostility [39]. Some studies have associated hostility and anger with a mixed state of depression and cited hostility as a marker of depression severity [40]. The results of correlation and regression showed that hostility was significantly positively correlated with depression—that is, a high level of hostility aggravated the degree of depression among drug addicts and non-addicts. Hostile affect had a greater impact on the level of depression in drug addicts. But it was worth noting that cynicism, aggressive response, hostile attitude, and social aversion were more impactful on the level of depression of non-addicts. It can be seen that hostility was more influential on the level of depression of non-addicts. It may also be that some young people prefer to use drugs to relieve psychological stress and reduce hostility. Therefore, more attention should be paid to the mental health status of children and adolescents in order to prevent them from taking the path of drug abuse due to psychological problems.

In this study, drug addicts were more depressed than non-addicts. This conclusion was consistent with previous study [16]. In other words, drug use increases the likelihood of depression [41]. Meanwhile, depression is a risk factor for developing addiction [20], and if unaddressed, it will relapse after treatment completion [22]. It can be concluded that depression and addiction are closely related and influence each other. This may be the reason why addicts become trapped in a vicious cycle of drug abuse and depression. As far as we know, people with a long history of drug abuse are typically labeled negatively and are discriminated against and excluded from society. These negative labels aggravate hostility, leading to extreme personality, social avoidance, or aggressive behaviors, and then produce a range of mental health problems, especially depression. Society should treat drug addicts equally, ensure the fairness of their rights, and reduce the discrimination and exclusion against them. In addition, more attention should be paid to the mental health of drug addicts, so that they can get rid of mental problems such as depression and return to society in a better way.

### Sense of life meaning among females drug addicts was higher than males, which was consistent with non-addicts

According to our results, females reported a better sense of life meaning than males, and male drug addicts were more depressed. These results of the study were also inconsistent with the conclusions of previous studies [26, 42–44]. The gender differences found in this study may be due to the different pressure experienced by male and female. In the harsh social competitive environment, males face greater social pressure [45], which leads to an increased psychological burden. Male drug addicts suffer more severe discrimination and injustice in society, which increases their hostility to society. In turn, intensifying social conflicts and disturbing public order lead to further prejudice against this group. Over time, the sense of life meaning for male drug addicts decreases. Therefore, we should pay more attention to male mental health, especially male drug addicts. When necessary, lectures on the meaning of life should be held to make them realize the importance of life meaning.

### Sense of life meaning’s partial mediating role in the relationship between hostility and depression

The results of the mediating effect model showed that sense of life meaning negatively predicted the level of depression. This result was consistent with a previous study on the negative correlation between sense of life meaning and depression [46]. Improving the sense of life meaning played a significant role in reducing depression. This was consistent with previous studies reporting that the sense of life meaning helped reduce the level of depression [31, 47].

Additionally, hostility negatively predicted the sense of life meaning for both drug addicts and non-addicts. However, the predictive variables in our mediation model were different for different groups. For drug addicts, the predictive variable was social aversion. The social aversion of drug addicts was a factor affecting depression. Some studies have shown that a lack of life meaning among drug addicts can lead to drug addiction [48], which can have negative effects on society. People with a long history of drug addiction are negatively labeled, and they will suffer more unfair treatment from society. Their participation in health care services is limited, and they often suffer humiliation and abuse when accessing services. One study found that many participants felt that they were treated unfairly or discriminated against for injecting drug use in healthcare [49]. When they feel hostility from society, they will become self-denying and lose confidence in their social interactions, which in turn lead to the loss of sense of life meaning. Drug addicts can generate social aversion or aggressive behaviors [50], which may lead to a range of mental health problems. Therefore, drug addicts need the care and help of society, and they should be provided with psychological counseling when necessary to relieve their hostility, which can reduce the likelihood of depression. All parties must make joint efforts to help drug addicts return to society smoothly. While for non-addicts, it was cynicism. Cynicism was an important factor affecting depression among non-addicts. In a fiercely competitive social environment, stress was an unavoidable problem for everyone. The study showed that high work stress was associated with cynicism and cynicism mediated the relationship between work stress and depression [51]. High-intensity work may lead to distrust and cynicism among employees, which in turn may be an influencing factor for mental illness. The finding suggested that higher life meaning was inversely related to psychological stress and that life meaning was a protective factor regarding stress-related reactions [52]. With the changes of time and the progress of technology, the generation of stress is inevitable. In this case, people should learn not only how to release their stress, but also how to maintain positive emotions and reduce the level of depression. The importance of enhancing life meaning is evident from the fact that it increases the sense of life meaning and reduces hostility and depression. Therefore, the sense of life meaning should be actively advocated.

## Limitations and directions for future research

Several limitations must be considered when interpreting the results of this study. First, the drug addicts recruited for this study are members of only two drug rehabilitation centers in Sichuan Province, which may limit the representativeness of the sample. Second, there are significant differences in age and BMI between the two groups, which reduce the comparability between the two groups in this study. Third, the sampling method may be the reason for the inconsistency of the conclusions with the previous. Fourth, this study adopted a cross-sectional design, which could not capture the psychological and emotional changes of individuals, such as hostility and depression.

For future research directions, some factors should be considered. First, in future studies, we should take into account the views of drug addicts on the number and duration of drug use, and minimize the intervention of drug factors in the study. Second, participants will be identified in the initial questionnaire as having a specific history of mental illness to ensure the accuracy of the study. Third, age should be taken into account in future studies and the role of age in the study should be analyzed. It is also important to control the consistency of demographic information and strengthen the comparability between groups when recruiting participants. Fourth, when analyzing the role of gender in research in future studies, it is also important to consider the differences that exist in gender itself, such as hormones and brain function. Finally, future research should enrich the sample by sampling a wider range of people and adopting a longitudinal study design to further confirm the influences of hostility and sense of life meaning on depression.

## Conclusions

This study provides evidence for a significant correlation between hostility and depression levels in both drug addicts and non-addicts, as well as evidence for the mediating role of the sense of life meaning between hostility and depression. Depression was more severe among drug addicts than among non-addicts. Higher levels of hostility lead to higher levels of depression, and the sense of life meaning, as a protective factor, can effectively reduce depression states. Our findings help to better understand the relationship between hostility and depression, and guide drug addicts and non-addicts to enhance their sense of life meaning in their lives and work, and promote their physical and mental health.

## Data Availability

The raw data supporting the conclusions of this article will be made available by the authors, without undue reservation.

## Abbreviations

CMI: Cook-Medley Hostility Scale
BDI: Beck Depression Inventory
MLQ: Meaning in Life Questionnaire
